# Piezoelectric Actuators in Smart Engineering Structures Using Robust Control

**DOI:** 10.3390/ma17102357

**Published:** 2024-05-15

**Authors:** Amalia Moutsopoulou, Markos Petousis, Nectarios Vidakis, Anastasios Pouliezos, Georgios E. Stavroulakis

**Affiliations:** 1 Department of Mechanical Engineering, Hellenic Mediterranean University Estavromenos Heraklion Crete, 714 10 Iraklio, Greece; amalia@hmu.gr (A.M.); vidakis@hmu.gr (N.V.); 2 Department of Production Engineering and Management, Technical University of Crete, Kounoupidianna, 731 00 Chania, Greece; tasos@dpem.tuc.gr (A.P.); gestavr@dpem.tuc.gr (G.E.S.)

**Keywords:** piezoelectric actuators, reduced order control, smart structure, robust control

## Abstract

In this study, piezoelectric patches are used as actuators to dampen structural oscillations. Damping oscillations is a significant engineering challenge, and the use of piezoelectric patches in smart structures allows for a reduction in oscillations through sophisticated control methods. This analysis involved H-infinity (H∞) robust analysis. H∞ (H-infinity) control formulation is a robust control design method used to ensure system stability and performance under disturbances. When applied to piezoelectric actuators in smart structures, H∞ control aims to design controllers that are robust to variations in system dynamics, external disturbances, and modeling uncertainties, while meeting specified performance criteria. This study outlines the piezoelectric effects and advanced control strategies. A structural model was created using finite elements, and a smart structural model was analyzed. Subsequently, dynamic loads were applied and oscillation damping was successfully achieved by employing advanced control techniques.

## 1. Introduction

Recently, there has been a significant focus on smart structures [1,2,3,4]. An intelligent structure is characterized by its ability to detect mechanical disruptions and respond automatically by minimizing oscillations [5,6,7,8,9]. This study introduces a smart structure featuring integrated actuators and sensors designed to dampen oscillations. The structure was analyzed under dynamic loads, such as wind forces, using finite element methods. Advanced examination methods such as robust control theory were employed in this study [10,11,12,13,14].

The development of control strategies for piezoelectric smart structures presents several challenges. To provide efficient and cost-effective active control, extensive research has been conducted on the use of piezoelectric materials in systems with dispersed parameters [13,14,15,16,17]. Utilizing distributed piezoelectric material-based sensors and actuators with adaptable properties enables the active regulation of dynamic systems [18,19,20,21,22,23]. This study explores the critical considerations that structural control engineers must address when devising reliable control methods. In the field of engineering, control theory is a commonly applied method for material optimization [9,12], as evidenced by previous studies [9,10,11,12,13,14,24]. Robust control techniques have been effectively utilized in intelligent materials [15,16,17,18,19,20,21,22,23,25]. In this context, we focused on the application of piezoelectric components. The discovery of piezoelectricity originated in 1880 when the Curie brothers (Pierre and Jacques) observed the generation of electric fields in quartz crystals subjected to mechanical forces. The term “piezo” originates from the Greek word meaning “press” [1,2,3,4,5,6,7,8,9].

Piezoelectric materials are pivotal components of smart structures because of their distinctive capability to transform mechanical energy into electrical energy, and inversely [1,2,3,4,5,6,7,8]. This distinctive property enables a wide range of applications of piezoelectric materials in smart structures, including sensing, actuation, and energy harvesting functionalities [25,26,27,28,29,30,31,32]. There are some key insights about piezoelectric materials in smart structures: for sensing functionality, piezoelectric materials are frequently utilized as sensors in smart structures to detect mechanical parameters, such as stress, strain, pressure, or vibrations; the use of piezo elements in the vibration measurement of objects with nonlinear characteristics, such as drones; and the use of piezoelectric elements for the vibration measurements of objects with unusual shapes and nonlinear characteristics has been presented in the literature [33,34]. These studies indicate new possibilities for the application of piezoelectric elements in the measurement of object vibrations.

When subjected to mechanical stress, a piezoelectric material generates an electric charge proportional to the applied stress. This characteristic makes piezoelectric sensors well-suited for monitoring structural health, identifying damage, or measuring dynamic loads within a structure [35,36,37,38,39]. Actuation Capabilities [35,36,37,38,39,40,41]: In addition to their sensing capabilities, piezoelectric materials are also deployed as actuators in smart structures. When an electrical signal is applied to a piezoelectric material, it undergoes mechanical deformation or vibrations. This deformation can be harnessed to control the shape, position, and damping properties of the structure. Piezoelectric actuators offer precise and responsive control, making them valuable for active vibration control, shape morphing, and structural adjustments in smart systems. Integration and Versatility [29,30,31,32,35]: Piezoelectric materials are highly adaptable and can be integrated into various structural elements, including beams, panels, and composites, without significantly altering the overall design or performance of the structure. This versatility allows the seamless incorporation of piezoelectric functionalities into existing or new smart structures, thereby enhancing their functionality, responsiveness, and overall performance.

In summary, piezoelectric materials play a pivotal role in smart structures by offering sensing, actuation, and energy harvesting capabilities [1,2,3,4,5,6,7,8]. Their unique properties make them indispensable components for enhancing structural monitoring, control, and energy efficiency in a wide range of applications. Piezoelectricity serves as a bridge between the electric and elastic fields, facilitating the evaluation of resilience, optimal placement, and structural modeling under uncertain conditions [35,36,37,38,39,40,41].

The reduction in vibrations under dynamic and unpredictable stresses constitutes a crucial engineering issue, and this challenge was investigated in our work. Vibrations play a critical role in systems of engineering due to the fact that they have been associated with material exhaustion, which can result in catastrophic failures and the premature failure of components [39,40,41,42,43,44]. This paper presents innovations in the control of piezoelectric structures. Comparative results are provided, and the complete suppression of oscillations is achieved. This has not been performed in any of the studies presented in the references. In this case, the oscillations are partially suppressed, as in previous works [15,16,17,29,30]. Advanced control techniques were used to completely suppress oscillations. Other studies have used simpler control techniques [16,17,30]. In our work, we use infinity control theory. In the present study, we achieved complete oscillation suppression using two methods of control, notably employing H∞ control. Initially, we modeled a smart structure featuring a combination of piezoelectric systems that act as sensors and actuators. Subsequently, we optimized their placement within the structure [41,42,43,44,45,46].

Simplifying the models enables the utilization of complex control methods, especially given that the presented controller is fairly satisfactory, specifically of the order of 36. All simulations were conducted using MATLAB v.13 (Mathworks, Natick, Massachusetts, United States) with proficient programming methodologies.

Our approach considers the modeling uncertainties and measurement noise, followed by the application of advanced control techniques. H∞ (H-infinity) control, which is known for its robustness against uncertainties, disturbances, and variations, was used in our system design. This control methodology specifically addresses the uncertainties stemming from the electromechanical properties of piezoelectric materials, system dynamics, external disturbances, and modeling inaccuracies [41,42,43,44,45]. In other studies, advanced control techniques were not used, but simpler choices were applied that did not yield such good results [16,17,30].

The core principle of H∞ control is structured singular value analysis, which is often referred to as μ analysis. This analysis quantifies and manages the uncertainties within a system by evaluating the structured singular value, denoted as the structural singular value, μ. The μ value represents the worst-case uncertainty magnitude capable of destabilizing the system. By optimizing the controller design to minimize μ, H∞ control ensures robustness within specified uncertainty bounds, making it suitable for systems such as those incorporating piezoelectric actuators in smart structures [41,42,43,44,45].

## 2. Materials and Methods

### 2.1. Piezoelectric Materials

Conventional electromechanical coefficients of piezoelectric materials, as measured by using standard methods or provided by manufacturers, lose their descriptive power when the electric field exceeds a specific nonlinearity threshold [46,47]. Both piezoelectric and dielectric factors increase as the applied electric field intensifies [46,47,48,49]. This field-dependent behavior was modeled based on the underlying mechanism driving such nonlinearity as well as the specific piezoelectric response pattern under investigation. The piezoelectric longitudinal response is described by Rayleigh’s law, which is derived from thermodynamic principles, in one of the models of field-dependent piezoelectric nonlinearity [47,48,49].

Similar modeling approaches have also been applied to describe the dielectric response: d_31_ E = d_i_ + a_cp_ E [46,47,48,49]. Parameter di represents the primary strain (or charge) piezoelectric connection factor, and αcp denotes the Rayleigh coefficient related to the converse piezoelectric (cp) effect. However, the linear correlation between the piezoelectric coupling coefficient, d, and the amplitude of the applied alternating current (AC) electric field, E, did not accurately match the experimental results for various soft piezoceramics exhibiting transverse response modes [46]. Consequently, an innovative mathematical approach was proposed in a previous study, wherein ceramics exhibited behavior similar to that of a hysteretic transducer. This model encompasses Rayleigh’s law as a special case study. In another study [47,48,49], it was postulated that the observed hysteretic changes in electromechanical properties stem from the field-dependent mechanical tension in the partially restricted crystallites of ceramics at the boundaries between domains. Additionally, using the electric pulse technique [48], it was discovered that irreversible alterations in remnant polarization occur even at electric fields significantly below the coercive threshold. This perspective contrasts with theories centered on 90° reorientation or tetragonal/rhombohedral phase boundary motion and argues that the high-field behavior of soft piezoceramics arises from hysteresis due to polarization reorientation (switching) [45,46,47,48,49].

### 2.2. Smart Structures

This analytical investigation explored the viability of integrating co-localized actuator pairs utilizing a transverse (d31)-mode piezoceramic (PZT G-1195) into beam coupons made of metallic (aluminum) and laminated composite materials (glass/epoxy and graphite/epoxy). Both surface-bonded and embedded configurations have also been examined [46,49].

The results indicate that the embedded graphite/epoxy composite smart beam with piezoceramics exhibited a linear response with increasing applied voltage. However, nonlinear deflection–voltage curves were observed in other smart beams. The aluminum beam featuring symmetrically surface-bonded actuator pairs demonstrated a reasonable model–test correlation, whereas the piezoceramic-embedded glass/epoxy composite smart beam showed a significant deviation from linearity.

In our simulation, we utilized piezoceramic (PZT G-1195) co-localized actuator pairs embedded in metallic (aluminum) and laminated composite (glass/epoxy and graphite/epoxy) beams [45,46,47,48,49].

### 2.3. Motion Equation of the Intelligent Structure

The beam equation for electrical and mechanical loading is given by [19,20,21,22,37]

(1) $$EI\frac{\partial^{4}y(t,x)}{\partial x^{4}}+\rho bAb\frac{\partial^{2}y(t,x)}{\partial t^{2}}=f_{m}\left(t,x\right)+f_{e}\left(t,x\right)$$

**Figure 1:**
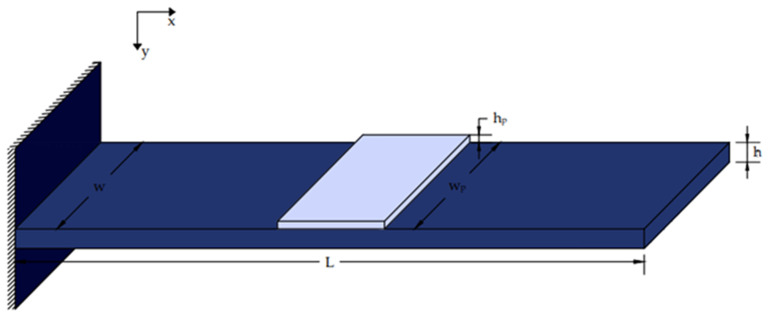
Beam with an installed piezoelectric patch.

**Figure 2:**
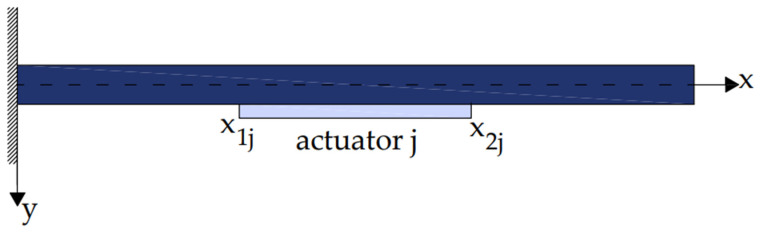
Piezoelectric section j inserted within the beam.

Figure 1 and Figure 2 illustrate a smart beam with an integrated piezoelectric actuator that outputs a mechanical force when given an electrical input [38,39,40,41,42,43,44]. The electric force *fe*(*t*,*x*) of the piezoelectric activator was determined as follows:

(2) $$f_{e}\left(t,x\right)=\frac{\partial^{2}M_{px}(t,x)}{\partial x^{2}}$$

where M_px_ indicates the torsion of the piezoelectric actuator.

The transfer function H indicates the placement of the piezoelectric patch Pzt on the beam. The torsion M_px_ is given by

(3) $$M_{px}(t,x)=C_{0}e_{pe}(t)[H\left(r-r_{1j}\right)-H\left(r-r_{2j}\right)]u_{j}(t)$$

where [18,19]

(4) $$C_{0}=EI\cdot K_{f}$$

(5) $$K_{f}=\frac{12EE_{p}hh_{p}(2h+h_{p)}}{16E^{2}h^{4}+EE_{p}\left(32h^{3}h_{p}+24h^{2}h_{p}^{2}+8hh_{p}^{3}\right)+E_{p}^{2}h_{p}^{4}}$$

The piezoelectric patch’s mechanical tension e_pe_(t) is determined by

(6) $$e_{pe}\left(t\right)=\frac{d_{31}}{h_{p}}u_{j}(t)$$

Thus, Equation (3) can be expressed as

(7) $$M_{px}(t,x)=C_{p}[H\left(r-r_{1j}\right)-H\left(r-r_{2j}\right)]u_{j}(t)$$

where

$$C_{p}=EIK_{f}\frac{d_{31}}{h_{p}}$$

After partial production in Equation (2), using (3), the electric force is given by

(8) $$f_{e}\left(t,x\right)=C_{p}u_{aj}\left(t\right)[\delta^{\prime }\left(r-r_{1j}\right)-\delta^{\prime }\left(r-r_{2j}\right)]$$

where

$$\int_{-\infty }^{\infty }\delta^{\left(n\right)}\left(t-\theta \right)\varphi \left(t\right)={\left(-1\right)}^{n}\varphi^{\left(n\right)}(\theta )$$

By applying Equations (1) and (8), the equation describing the response of the smart beam to the vertical dynamic disturbance *q*_0_(*t*) and the electrical dynamic force resulting from the piezoelectric patch is derived as follows:

(9) $$EI\frac{\partial^{4}y(t,x)}{\partial x^{4}}+\rho bAb\frac{\partial^{2}y(t,x)}{\partial t^{2}}=q_{0}\left(t\right)+C_{p}u_{j}\left(t\right)[\delta^{\prime }\left(r-r_{1j}\right)-\delta^{\prime }\left(r-r_{2j}\right)]$$

**Figure 3:**
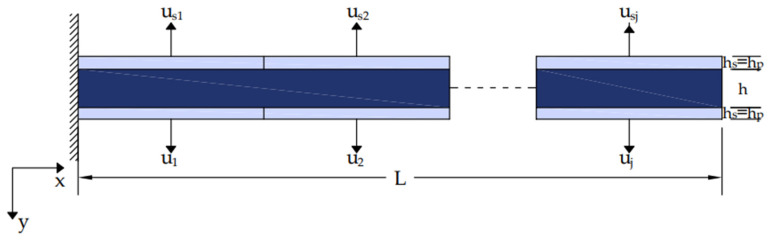
Smart beam equipped with integrated piezoelectric actuators and sensors.

For j similar piezoelectric (Figure 3) Equation (9) transforms to

(10) $$EI\frac{\partial^{4}y(t,x)}{\partial x^{4}}+\rho bAb\frac{\partial^{2}y(t,x)}{\partial t^{2}}=q_{0}\left(t\right)+C_{p}u_{j}\left(t\right)\sum_{i=1}^{j}\left[\delta^{\prime }\left(r-r_{1j}\right)-\delta^{\prime }\left(r-r_{2j}\right)\right]$$

### 2.4. Modeling

**Figure 4:**
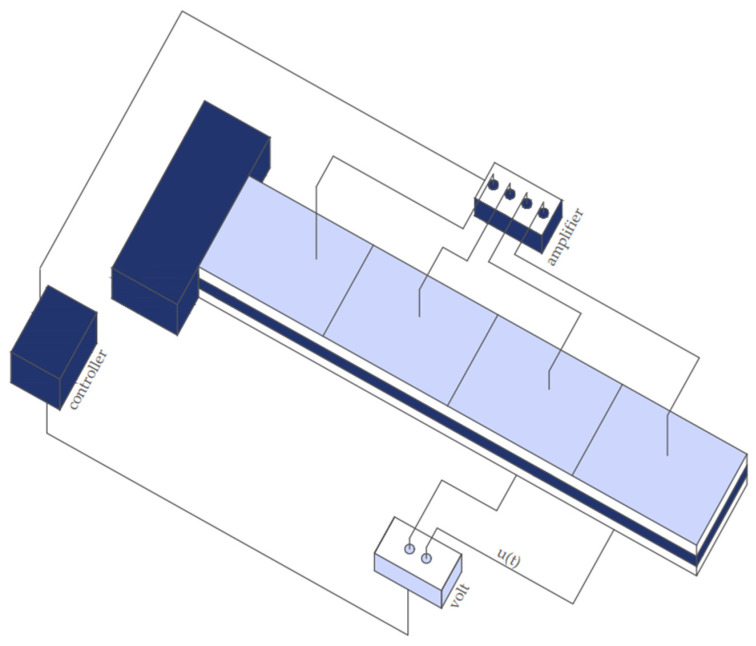
Positions 2 and 4 are where the actuators are alternately positioned.

This study focuses on reducing oscillations through the application of piezoelectric materials and complex control methods. Specifically, the placement of the piezoelectric actuators was considered. The actuators in Figure 4 are positioned throughout the beam, covering all positions labeled 1, 2, 3, and 4.

The system’s dynamical description is provided by [18,19,20,21,22,23]

(11) $$M\ddot{q}(t)+D\dot{q}(t)+Kq(t)\;=\;fm(t)\;+\;fe(t)$$

where fm represents the global external loading mechanical vector, K is the global stiffness matrix, M is the global mass matrix, D is the viscous damping matrix, and fe is the global control force vector resulting from electromechanical coupling effects. The rotations wi and transversal deflections ψi comprise the independent variable q(t), or

(12) $$q\left(t\right)=\left[\begin{array}{c} w_{1}\\ \psi_{1}\\ \vdots \\ w_{n}\\ \psi_{n} \end{array}\right]$$

where n indicates the number of finite elements used in the analysis [36,37,38,39]. Let us convert (as is customary) to a state-space control representation [11,12,13,14,15,16,17].

$$x\left(t\right)=\left[\begin{array}{c} q(t)\\ \dot{q}(t) \end{array}\right]$$

$$=\left[\begin{array}{c} 0_{2n\times n}\\ M^{-1}(f_{m}\left(t\right)+f_{e}(t) \end{array}\right]+\left[\begin{array}{c} \dot{q}(t)\\ -M^{-1}D\dot{q}\left(t\right)-M^{-1}Kq(t) \end{array}\right]$$

$$=\left[\begin{array}{c} 0_{2n\times n}\\ M^{-1}(f_{m}+f_{e})(t) \end{array}\right]+\left[\begin{array}{cc} \begin{array}{c} 0_{2n\times 2n}\\ -M^{-1}K \end{array} & \begin{array}{c} I_{2n\times 2n}\\ -M^{-1}D \end{array} \end{array}\right]\left[\begin{array}{c} q(t)\\ \dot{q}(t) \end{array}\right]$$

(13) $$=\left[\begin{array}{c} 0_{2n\times n}\\ M^{-1}f_{m}(t) \end{array}\right]+\left[\begin{array}{c} 0_{2n\times n}\\ M^{-1}f_{e}(t) \end{array}\right]+\left[\begin{array}{cc} \begin{array}{c} 0_{2n\times 2n}\\ -M^{-1}K \end{array} & \begin{array}{c} I_{2n\times 2n}\\ -M^{-1}D \end{array} \end{array}\right]\left[\begin{array}{c} q(t)\\ \dot{q}(t) \end{array}\right]$$

In addition, *fe*(*t*) is designated as where (2n × n) is the piezoelectric force of a unit placed on a suitable actuator.

(14) $$Fe\left(t\right)=\left[\begin{array}{cc} \begin{array}{cc} \begin{array}{c} 0\\ cp\\ \begin{array}{c} 0\\ \begin{array}{c} 0\\ 0\\ \begin{array}{c} 0\\ \begin{array}{c} 0\\ 0 \end{array} \end{array} \end{array} \end{array} \end{array} & \begin{array}{c} 0\\ -cp\\ \begin{array}{c} 0\\ \begin{array}{c} cp\\ 0\\ \begin{array}{c} 0\\ \begin{array}{c} 0\\ 0 \end{array} \end{array} \end{array} \end{array} \end{array} \end{array} & \begin{array}{cc} \begin{array}{c} 0\\ 0\\ \begin{array}{c} 0\\ \begin{array}{c} -cp\\ 0\\ \begin{array}{c} cp\\ \begin{array}{c} 0\\ 0 \end{array} \end{array} \end{array} \end{array} \end{array} & \begin{array}{c} 0\\ 0\\ \begin{array}{c} 0\\ \begin{array}{c} 0\\ 0\\ \begin{array}{c} -cp\\ \begin{array}{c} 0\\ cp \end{array} \end{array} \end{array} \end{array} \end{array} \end{array} \end{array}\right]$$

where u denotes actuator voltage. Finally, d(t) = fm(t) denotes a disturbance vector. Then,

$$\dot{x}\left(t\right)=\left[\begin{array}{cc} 0_{2n\times 2n} & I_{2n\times 2n}\\ -M^{-1}K & -M^{-1}D \end{array}\right]x\left(t\right)+\left[\begin{array}{c} 0_{2n\times n}\\ M^{-1}F_{e}^{*} \end{array}\right]u\left(t\right)+\left[\begin{array}{c} 0_{2n\times 2n}\\ M^{-1} \end{array}\right]d\left(t\right)$$

=Ax(t)+Βu(t)+Gd(t)

$$=\mathrm{Ax}(t)+[B\;G]\;\left[\begin{array}{c} u(t)\\ d(t) \end{array}\right]$$

(15) $$=\mathrm{Ax}(t)+\tilde{B}\tilde{u}(t),$$

Using the output equation (only displacements are measured), this can be improved.

y(t) = [x1(t) x3(t) … xn−1(t)]T = Cx(t)

where

C = [1 0 0…0; −1 0 1 
0…0; 0 0 −1 0 1 …0; 0 0 0 0 −1 0 1 …0]

The piezoelectric effect converts mechanical stress into strain, and strain into mechanical stress. This is the basis for the suppression of oscillations achieved in this work [4,5,6,7,8].

**Figure 5:**
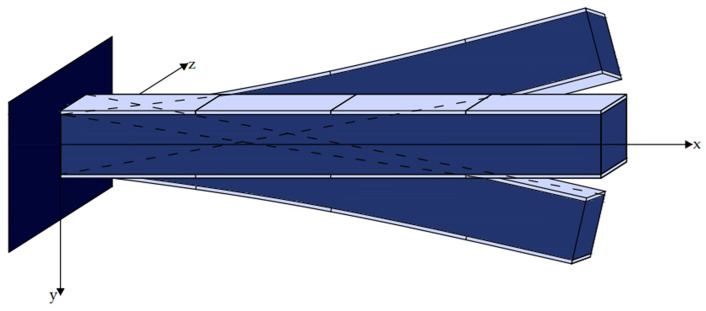
The actuators were placed across the beam.

In our simulation, we used co-localized actuator pairs with piezoceramic (PZT G-1195) in both metallic (aluminum) and laminated composite (glass/epoxy, graphite/epoxy) beams (Figure 5). The parameters of the smart beam are presented in Table 1.

### 2.5. Robustness Issues

H∞ control stands out because it explicitly considers the most detrimental impact caused by unknown disturbances and noise within a system. Theoretically, it is feasible to design an H∞ controller that is robust against a predetermined level of modeling inaccuracies. However, this potential is not always feasible, as will be elaborated upon later [41,42,43,44,45].

**Figure 6:**
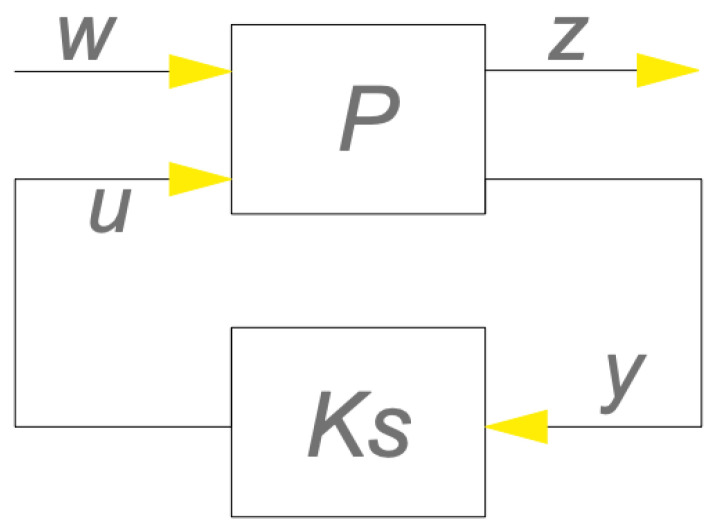
Block diagram of the smart structure.

**Figure 7:**
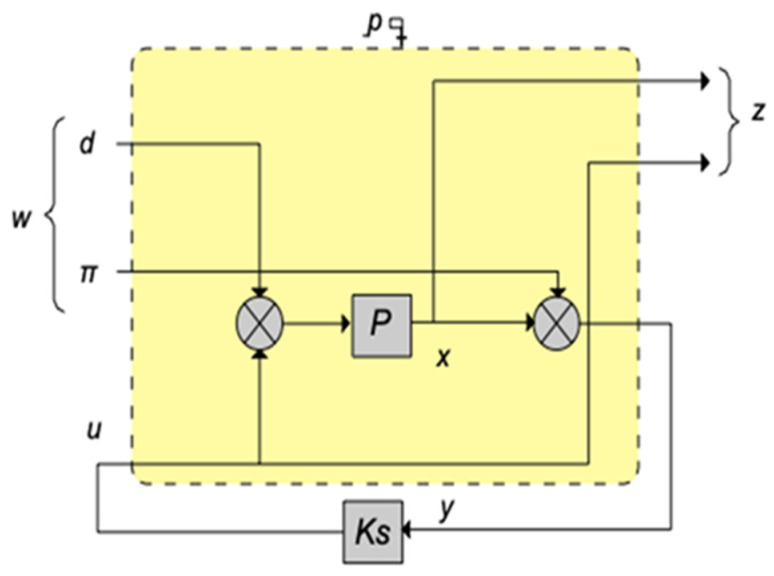
A more analytical block diagram of the smart structure.

Figure 6 and Figure 7 show the block diagrams of the intelligent beam system. Here, Ks represents the controller, w signifies the inputs, including disturbances and noise (n), z represents the output state vectors, P denotes the smart beam itself, and u signifies the applied control force [39,40,41,42,43,44,45].

We proceed with the following methodology to account for the uncertainty in matrices M and K.

K = K_0_(I + k_p_I_2n×2n_δ_K_) M = M_0_(I + m_p_I_2n×2n_δ_M_) (16)

Furthermore, since D = 0.0005(K + M), a suitable form for D is

D = 0.0005[K_0_(I + k_p_I_2n×2n_δ_K_) + M_0_(I + m_p_I_2n×2n_δ_M_)] = D_0_ = 0.0005K_0_ + 0.0005M_0_ D_0_ + 0.0005[K_0_k_p_I_2n×2n_δ_K_ + M_0_m_p_I_2n×2n_δ_M_] (17)

The damping matrix (D) characterizes the damping properties of the structure, with damping typically being a small proportion compared to the mass and stiffness matrices. Experimental investigations have determined this proportion to be 0.0005 for both mass and stiffness matrices, as referenced in [27]. Notably, lower damping values make it more difficult to effectively dampen the vibrations of a structure, as discussed in [26,27,28].

However, it is generally understood that

D = α K + β M

The structural damping matrix D is expressed as a linear combination of the mass matrix (M) and the stiffness matrix (K), and is commonly referred to as Rayleigh damping. In this framework, the coefficients α and β are determined based on the analysis of the first and second normal modes of vibration, with both coefficients typically set at 0.0005. Therefore, the formulation of D can be structured in a manner similar to how the stiffness matrix (K) and mass matrix (M) are typically represented as follows:

D = D_0_ (I + d_p_I_2n×2n_δ_D_) (18)

We incorporated uncertainty into the relevant matrices by introducing proportional deviations. This method of addressing uncertainty is notably effective in our scenario, given that length measurements can be performed with high accuracy. Uncertainty primarily stems from specific terms rather than the core matrices themselves. In this context, the following assumptions were made.

(19) $$║\Delta {║}_{\infty }\;\overset{\mathrm{def}}{=}{\left\|\left[\begin{array}{cc} {Ι}_{n\times n}\delta_{Κ} & 0_{n\times n}\\ 0_{n\times n} & {Ι}_{n\times n}\delta_{Μ} \end{array}\right]\right\|}_{\infty }<1$$

Thus, *m_p_* and *k_p_* were employed to adjust the proportion value, with nominal values denoted by the subscripts of zero.

(It is urged that for matrix A_n×m_, the norm is determined via ║A║_∞_ = $\underset{1\leq j\leq m}{max}\sum_{j=1}^{n}\left|a_{ij}\right|$)

Taking these specifications into consideration, Equation (13) changes to

(20) $$M_{O}\left(I+m_{p}I_{2nx2n}\delta_{Μ}\ddot{q}\left(t\right)\right)+Ko\left(I+k_{p}I_{2nx2n}\delta_{K}q\left(t\right)\right)+[D+0.0005\left[Kok_{p}I_{2x2}\delta_{K}+{M_{O}m}_{p}I_{2x2}\delta_{Μ}\right]\dot{q}\left(t\right)+f_{m}\left(t\right)+f_{e}(t)\;\Rightarrow M_{O}\ddot{q}\left(t\right)+D_{O}\dot{q}\left(t\right)+K_{O}q\left(t\right)=-\left[M_{O}m_{p}I_{2nx2n}\delta_{Μ}\ddot{q}\left(t\right)+0.0005\left[Kok_{p}I_{2x2}\delta_{K}+{M_{O}m}_{p}I_{2x2}\delta_{Μ}\right]\dot{q}\left(t\right)+{K_{O}k}_{p}I_{2nx2n}\delta_{K}q\left(t\right)\right]+f_{m}\left(t\right)+f_{e}\left(t\right)\;\Rightarrow M_{O}\ddot{q}\left(t\right)+D_{O}\dot{q}\left(t\right)+K_{O}q\left(t\right)=\tilde{D}q_{u}\left(t\right)+f_{m}\left(t\right)+f_{e}(t)$$

where

$$\begin{array}{c} q_{u}\left(t\right)=\left[\begin{array}{c} \ddot{q}(t)\\ \dot{q}(t)\\ q(t) \end{array}\right]\\ \tilde{D}=-\left[\begin{array}{cc} M_{0}m_{p} & K_{0}k_{p} \end{array}\right]\left[\begin{array}{cc} I_{2n\times 2n}\delta_{Μ} & 0_{2n\times 2n}\\ 0_{2n\times 2n} & I_{2n\times 2n}\delta_{Κ} \end{array}\right]\left[\begin{array}{ccc} I_{2n\times 2n} & 0.0005I_{2n\times 2n} & 0_{2n\times 2n}\\ 0_{2n\times 2n} & 0.0005I_{2n\times 2n} & I_{2n\times 2n} \end{array}\right]=\\ =G_{1}\cdot \Delta \cdot G_{2} \end{array}$$

(21) $$G1=-\left[\begin{array}{cc} M_{0}m_{p} & K_{0}k_{p} \end{array}\right],\;\;\;\;\;\;\;\;G2=\left[\begin{array}{ccc} I_{2n\times 2n} & 0.0005I_{2n\times 2n} & 0_{2n\times 2n}\\ 0_{2n\times 2n} & 0.0005I_{2n\times 2n} & I_{2n\times 2n} \end{array}\right]$$

Expressing Equation (7) in state space form yields

(22) $$\dot{x}\left(t\right)=\left[\begin{array}{cc} 0_{2n\times 2n} & I_{2n\times 2n}\\ -M^{-1}K & -M^{-1}D \end{array}\right]x\left(t\right)+\left[\begin{array}{c} 0_{2n\times n}\\ M^{-1}f_{e}^{*} \end{array}\right]u\left(t\right)+\left[\begin{array}{c} 0_{2n\times 2n}\\ M^{-1} \end{array}\right]\left(t\right)+\left[\begin{array}{c} 0_{2n\times 6n}\\ M^{-1}G_{1}\cdot \Delta \cdot G_{2} \end{array}\right]q_{u}\left(t\right)$$

(23) $$=Ax(t)+Βu(t)+Gd(t)+G_{u}G_{2}q_{u}(t)$$

Equations (11) and (12) represent the dynamic equations of motion for the structure, incorporating the mass and stiffness properties determined using the finite element method. These equations are formulated in the state-space domain, allowing us to derive the dynamic response of the structure, that is, how it oscillates both with and without control mechanisms.

By introducing parameters such as Kp and mp to alter the mass and stiffness registers, we can simulate changes in the initial conditions of the structure. This enabled us to model scenarios in which the structure was damaged or experienced shifts in its initial condition. In this methodology, we treat the uncertainty in the original matrices as an additional input representing the modeling uncertainty. These equations incorporate the concept of integrating active and adaptable control strategies into smart structures, allowing them to respond effectively to dynamic loads and changing environmental conditions. However, devising efficient control algorithms and systems capable of optimally reacting to external forces and structural states remains a significant challenge. Temperature changes change the displacements and in the hyperstatic carriers, the intensive state of the structure. However, in this application, while there is a production of electrical voltages, there is no temperature change. Temperature can also influence the frequency response of piezoelectric materials. Temperature changes can shift the resonance frequency of piezoelectric devices, which is crucial for applications such as ultrasonic transducers.

Engineers and researchers working with piezoelectric materials must consider these temperature effects to ensure the reliable and accurate performance of devices under different operating conditions. Techniques, such as temperature compensation and material selection based on the intended temperature range, are commonly employed to mitigate these effects.

## 3. Results and Discussion

### 3.1. Results

The following are the simulations of smart construction. In all simulations, we considered wind charging, which was simulated using a sinusoidal charge.

Certainly, here are the mathematical relationships commonly used to model wind forces in mechanical simulations [18,19,20,21,22,23,25,26]:

The uniform wind load g is typically calculated using the following formula:

*d*(*t*) = 0.5 × *CP* × *ρ* × *V*(*t*) (24)

where

*CP* is the pressure coefficient (dimensionless), *CP* = 1.5

*ρ* is the air density (kg/m³), and

where *V*(*t*) is wind speed (m/s).

Dynamic wind loads consider the dynamic response of a structure to varying wind speed and direction over time. This is often modeled using differential equations that describe the motion of a structure under dynamic wind forces.

In our simulation, *V*(*t*) = 10 sin(t), and in relation (23),

d(t) = 0.5 × *C*p × ρ × V(t) (25)

These mathematical relationships are fundamental for accurately modeling wind forces in mechanical simulations and predicting the structural response under different wind conditions.

Therefore, nominal values are represented by zero subscripts, and *m_p_* and *k_p_* are used to scale the value of the proportion.

By introducing parameters such as Kp and mp to alter the mass and stiffness registers, we can simulate changes in the initial conditions of the structure. This enabled us to model scenarios in which the structure was damaged or experienced shifts in its initial condition. Using this methodology, we treated the uncertainty in the original matrices as a further parameter representing modeling uncertainty.

**Figure 8:**
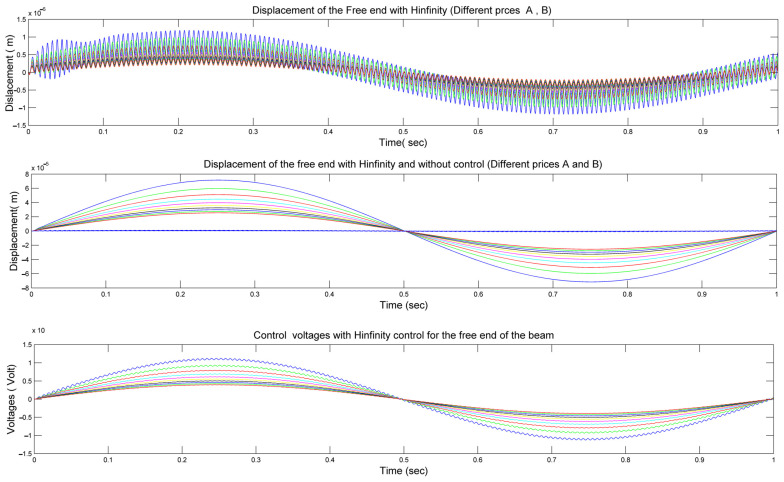
(**Upper diagram**): Displacement (without control) of the free end of our smart beam in matrices A and B by changing mp and kp. (**Middle diagram**): Displacement without control (colored foul lines) and with control (different blue lines) of the free end of our smart beam in matrices A and B (different mp, kp). (**Lower diagram**): Control voltages for different prices of our smart beam in matrices A and B (different mp and kp).

In Figure 8, we present a comprehensive analysis of the displacement behavior at the free end of our smart beam under varying conditions of parameters *mp* and *kp*, as represented by matrices A and B in Equation (23).

The upper diagram in Figure 8 illustrates the displacement without control, showing the outcomes with changes in *mp* and *kp*. This depiction helps us to understand the inherent behavior of a smart beam under different configurations.

In the middle diagram, we delve deeper into the displacement scenario by contrasting the outcomes without control (depicted by colored foul lines) with those achieved with control (depicted by various blue lines). Notably, when H-infinity control is applied, the displacements approach nearly zero, indicating that the smart beam remains in equilibrium according to the principles of H-infinity control theory.

Finally, the lower diagram in Figure 8 provides insights into the control voltages required for different configurations of our smart beam, again considering variations in *mp* and *kp*, as per Equation (23). Notably, the maximum control voltages observed were 150 Volts lower than the maximum voltages of the PZT patches, which were rated at 500 Volts.

Figure 8 presents a detailed exploration of the displacement and control dynamics of our smart beam under varying parameters, shedding light on the efficacy of H-infinity control in maintaining equilibrium and minimizing control voltages within safe operational limits.

**Figure 9:**
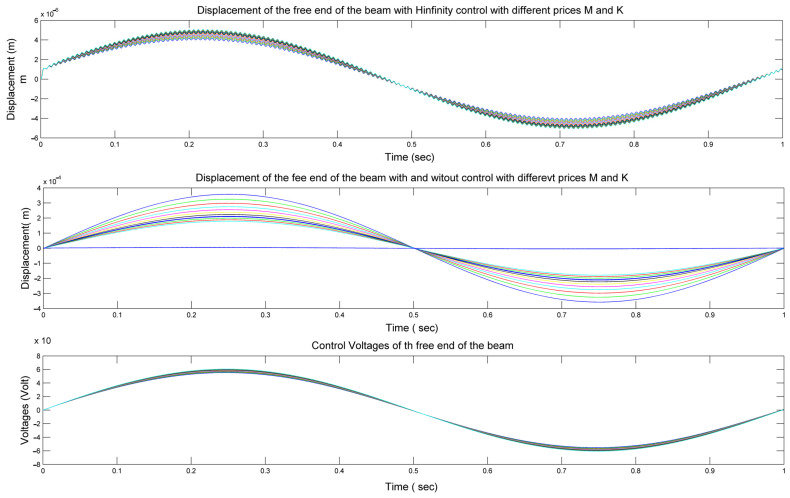
(**Upper diagram**): Displacement (without control) of the free end of our smart beam in matrices M and K by changing mp and kp. (**Middle diagram**): Displacement without control (colored foul lines) and with control (different blue lines) of the free end of our smart beam in matrices M and K by changing mp and kp. (**Lower diagram**): Control voltages for different prices of our smart beam in matrices M and K (different values of mp and kp).

Figure 9 provides a detailed analysis of the displacement behavior at the free end of our smart beam under varying conditions of parameters *mp* and *kp*, as represented by matrices M and K in Equation (23).

In the upper diagram of Figure 9, we observe the displacement without control, illustrating how changes in *mp* and *kp* affect the behavior of the smart beam.

In the middle diagram, we delve deeper into the displacement scenario by comparing outcomes without control (depicted by colored foul lines) with those achieved with control (depicted by various blue lines). Notably, the inclusion of H-infinity control results in displacements approaching zero, indicating that the smart beam maintains equilibrium in accordance with H-infinity control principles.

The lower diagram in Figure 9 offers insights into the control voltages necessary for different configurations of our smart beam, again considering variations in *mp* and *kp*, as per Equation (23). Notably, the maximum control voltages observed were 150 Volts lower than the maximum voltages of the PZT patches, which were rated at 500 Volts.

Figure 9 provides a comprehensive view of the displacement and control dynamics of our smart beam under varying parameters, highlighting the effectiveness of H-infinity control in achieving equilibrium and minimizing the control voltages while ensuring operational safety. In Figure 8 (Upper diagram) and Figure 9 (Upper diagram) (in the first diagrams), the diagrams show the changes in the displacements for different values of the stiffness mass matrices A and B with the application of infinity intelligent control. They are zoomed-in images of the profiles shown in Figure 8 (Middle diagram) and Figure 9 (Middle diagram). It should be noted that in Figure 8 and Figure 9, we do not have phenomena such as temperature change, which will be examined in future work.

Piezoelectric materials are fascinating because they exhibit a unique property: when mechanical stress is applied to them, they generate an electric charge; conversely, when an electric field is applied, they undergo mechanical deformation. This phenomenon makes them highly useful in various applications such as sensors, actuators, and energy-harvesting devices.

The piezoelectric effect is affected by temperature in several ways.

The piezoelectric effect is closely related to the elasticity of a material. Temperature changes can alter the elasticity of a material, thereby affecting its piezoelectric properties. Different piezoelectric materials have different temperature coefficients of elasticity, indicating the extent to which their piezoelectric response changes with temperature.

Temperature can also influence the frequency response of piezoelectric materials. Temperature changes can shift the resonance frequency of piezoelectric devices, which is crucial for applications such as ultrasonic transducers.

Engineers and researchers working with piezoelectric materials must consider these temperature effects to ensure the reliable and accurate performance of devices under different operating conditions. Techniques, such as temperature compensation and material selection based on the intended temperature range are commonly employed to mitigate these effects.

**Figure 10:**
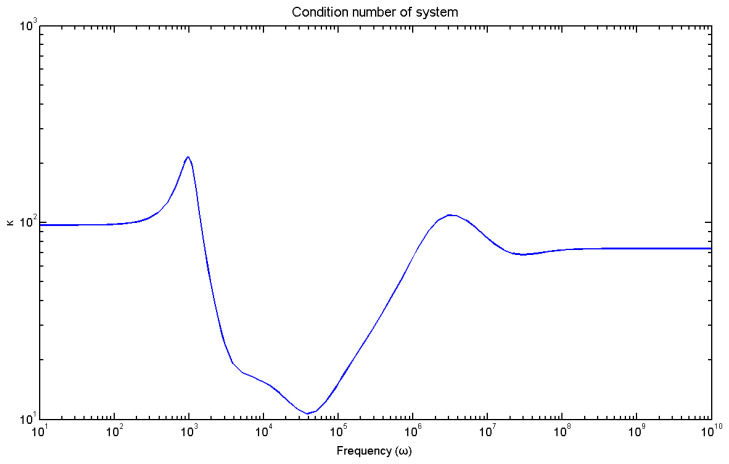
Condition number of our system.

Subsequently, the results for the frequency field are presented. Figure 10 shows the system condition number of our system, which is the ratio of the minimum to the maximum eigenvalues of our smart system. The index of our system is high at low frequencies. The proposed system was controllable and observable.

**Figure 11:**
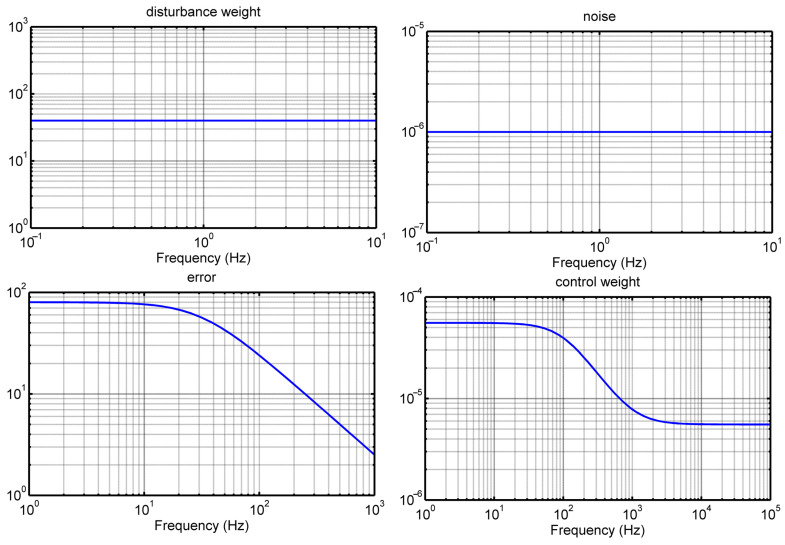
Bode diagram of the smart system.

In the realm of frequency analysis, we delve into Figure 11, which illustrates the diagonal elements of weighting matrices. These matrices were the result of extensive testing aimed at ensuring the robustness and dependability of the currently employed controller. Notably, our controller boasts an order of 36, and the maximum eigenvalue *γ* = 0.074 signifies a critical characteristic of our control system.

**Figure 12:**
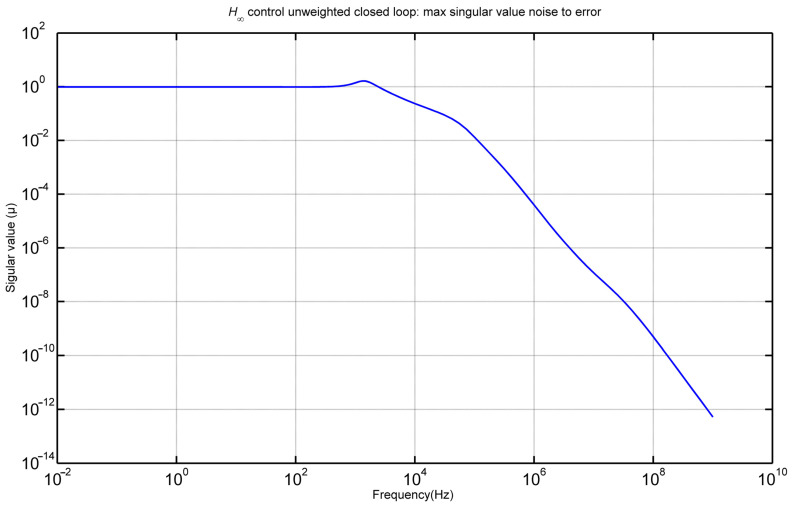
Max singular value noise to error.

Figure 12 shows the maximum singular value noise with respect to the error. The diagram provides a visual representation of the maximum singular values of the transfer functions associated with the closed-loop unstable system, focusing on the original values of particular interest. Upon closer examination, it is evident that noise has a limited influence on the error, particularly at frequencies exceeding 1000 Hz. This observation underscores the robustness of the system and its ability to maintain stability and accuracy even in the presence of external disturbances or noise at higher frequencies.

### 3.2. Discussion

Piezoelectric materials play a crucial role in smart structures because they can convert electrical energy from mechanical energy, and vice versa. These materials possess unique features that allow for them to be used in a variety of smart structures, such as sensors, actuators, and energy harvesters [1,2,3,4,5,6,7,8,9]. Here are some key points about piezoelectric materials in smart structures: Piezoelectric materials can be used as sensors to detect mechanical stress, strain, pressure, or vibrations in smart structures [10,11,12,13,14,15,16]. When mechanical stress is applied to a piezoelectric material, it generates an electrical charge proportional to the applied stress. This property makes piezoelectric sensors ideal for monitoring structural health, detecting structural damage, or measuring dynamic loads. In addition to smart structures, piezoelectric materials are often employed as actuators to induce mechanical deformations or vibrations in response to applied electrical signals [27,28,29,30,31,32,33,34,35,36,37]. When an electric field is applied to a piezoelectric material, it undergoes mechanical deformation that can be used to control the shape, position, or damping of the structure. Piezoelectric actuators are used in applications such as vibration control, shape morphing, and precise positioning [18,19,20,21,22,23,25,26,27]. In summary, piezoelectric materials play a vital role in enhancing the functionality, efficiency, and performance of smart structures by enabling sensing and actuation [28,29,30,31,32,33]. Their integration and optimization are key areas of research and development aimed at advancing the field of smart structures for various engineering applications. This study focuses on employing a robust control theory to minimize vibrations in smart structures. Such structures integrate advanced sensing, actuation, and control systems to monitor and mitigate structural vibrations actively. Sensors track parameters such as vibrations and displacements, whereas actuators (often piezoelectric) apply control forces based on sensor feedback. Robust control methods, such as H∞ control, μ-synthesis, and model predictive control (MPC), are key to managing uncertainties and dynamic variations effectively. Understanding a structure’s dynamics via techniques such as finite element modeling (FEM) and modal analysis is crucial for developing precise control strategies [38,39,40,41]. These goals include reducing resonance, damping unwanted vibrations, improving stability, and ensuring structural safety.

In this study, we leveraged the capabilities of piezoelectric materials, both as sensors and actuators, to effectively dampen oscillations. This approach necessitates the use of a finite element formulation for structural analysis and a robust control theory for system stability and performance enhancement. Specifically, we employ the H-infinity (H∞) control theory, which is a robust control design methodology [18,27,41,42,43,44,45].

H∞ control is adept at ensuring system stability and performance even in the face of uncertainties and disturbances. When applied to piezoelectric actuators within smart structures, H∞ control aims to design controllers that exhibit robustness against variations in system dynamics, external disturbances, and modeling uncertainties. This approach is critical for meeting specified performance criteria and ensuring the reliable and effective operation of smart structures in real-world applications.

The key steps and considerations for applying the H∞ control formulation to piezoelectric actuators in smart structures are as follows [18,19,20,21,22,23,25,26,27,41,42,43,44,45]:

1. System Modeling: The first step is to develop an accurate mathematical model of the smart structure dynamics, including the piezoelectric actuator, structural components, sensors, and external disturbances. The model captured the electromechanical coupling of the piezoelectric material, structural dynamics, and feedback loops.
2. Uncertainty Description: H∞ control addresses uncertainties in a system model. These uncertainties can arise from modeling errors, parameter variations, environmental changes, or disturbances. It is essential to quantify these uncertainties and represent them within a control design framework.
3. Performance Specifications: Performance criteria for a smart structure system are defined. This includes stability requirements, tracking accuracy, disturbance rejection, bandwidth limitations, and robustness margins. These specifications guide the design of the H∞ controller to ensure that the system satisfies the desired performance objectives under various conditions.
4. Controller Design: Utilize H∞ control synthesis techniques to design a robust controller that minimizes the effects of uncertainties and disturbances on system performance while satisfying performance specifications. H∞ controllers are typically designed based on a structured singular value (μ) optimization framework that aims to minimize the worst-case sensitivity of the system.
5. Controller Implementation: The designed H∞ controller is implemented on the smart structure system and integrated with the piezoelectric actuator control loop. This involves tuning controller parameters, setting up feedback loops, and interfacing sensors and actuators to achieve the desired control behavior.

By applying the H∞ control formulation to piezoelectric actuators in smart structures, engineers can design robust and high-performance control systems that can effectively manage uncertainties, disturbances, and variations in the system dynamics. This approach enhances the reliability, stability, and functionality of smart structures in various applications such as vibration control, shape morphing, and structural health monitoring.

Many researchers have been engaged in the application of modern methods to the development of new technologies and the application of sensors and actuators [49,50,51,52]. This is our future research, as well as the introduction of technical intelligence into our research.

## 4. Conclusions

This research utilized robust control theory to reduce vibrations in a smart structure. Smart structures integrate advanced sensing, actuation, and control systems to actively manage and mitigate structural vibrations by monitoring parameters, such as vibrations and displacements. Actuators, particularly piezoelectric actuators, apply control forces based on sensor feedback to induce mechanical deformations. Robust control algorithms, such as H∞ control, μ-synthesis, and model predictive control (MPC), are crucial for effectively managing vibrations and handling uncertainties, disturbances, and dynamic variations. Understanding the structural dynamics via techniques such as finite element modeling (FEM), modal analysis, and system identification helps develop precise control strategies. The objectives of smart structures for vibration reduction include minimizing resonance, damping unwanted vibrations, improving stability, and ensuring structural integrity and safety.

Designing a smart structure for reducing vibrations with robust control involves a multidisciplinary approach that combines structural engineering, control theory, sensing technologies, and advanced algorithms to achieve enhanced performance, safety, and reliability in dynamic environments.

In this study, we successfully achieved the complete suppression of oscillations in intelligent systems with H-infinity control. We consider modeling uncertainties and measurement noise and then apply advanced control techniques. Our research results are shown in both time and frequency domains. The primary advantages of this study are as follows:

1. Modeling Intelligent Structures for Control in Oscillation Suppression
  - We developed a mathematical model for intelligent structures that allows for the effective control of oscillations.
2. Handling Uncertainties in Dynamic Loading.
  - Our approach addresses the uncertainties arising from dynamic loading conditions and ensures robust control performance.
3. Measurement Noise management
  - We accounted for measurement noise in the system, thereby enhancing the accuracy and reliability of our control strategy.
4. Selection of Optimal Weights for Suppression of Oscillation
  - Through appropriate weighting functions, we achieved the complete suppression of oscillations and optimized the control performance.
5. Analysis of Time and Frequency Domains
  - Our results are presented and analyzed comprehensively in both the time and frequency domains, providing a thorough understanding of control performance.
6. Incorporation of Uncertainties in the Mathematical Model of the Structure
  - Uncertainties were introduced into the mathematical model of the structure, making our approach more robust and adaptable to real-world conditions.

Overall, our study contributes to the advancement of control strategies in smart structures by effectively suppressing oscillations, considering uncertainties, and optimizing control performance through advanced techniques. Future investigations should focus on two primary avenues. First, the application of these control strategies to actual intelligent structures within an experimental framework is the focal point. Second, different control methodologies aimed at suppressing structural noise and vibration will be explored. In addition, the application of technical intelligence to suppress oscillations will be the subject of future research.

## Figures and Tables

**Table 1:** Parameters of the smart beam.

Parameters	Values
L, for beam length	1.20 m
W, for beam Width	0.08 m
h, for beam thickness	0.02 m
ρ, for beam density	1700 kg/m^3^
E, for Young’s modulus of the beam	1.6 × 10^11^ N/m^2^
bs, ba, for Pzt thickness	0.002 m
d_31_ the Piezoelectric constant	280 × 10^−12^ m/V

## Data Availability

Data presented in this study are available upon request from the corresponding author (due to privacy).

## Nomenclature

M:	Mass matrix	ψ_i_(t):	Displacement deflection
K:	Stiffness matrix	x(t):	The state vector of our system
D:	Viscous damping matrix	y(t):	Output vector of our system
fe(t) fm(t)	Piezoelectric force External mechanical force	d_31_:	Piezoelectric constant
n:	Number of nodes in finite element formulation	*C_p_*, *C_o_* *Kf*	Piezoelectric constant
u(t):	Control voltages of actuators (control vector)	*K*(*s*):	*Hinfinity* Controller of the system
Fe:	Matrix with piezoelectric constant	E	The young modulus of the beam
w_i_(t):	Rotation deflection	*P*:	Augment plant of the smart system
*μ*:	Singular value	*e*(t):	The error of the system
d(t):	Disturbances of the system	*n*(t):	Noise of the system
*A*, *B*	Matrices of our system	*D*, *G*-*K:*	*D*–*K* interaction in the frequency domain
*w*	Inputs of the smart systems (external disturbance, noise)	z	Outputs of the smart system (control vector, output vector)
W_n_:	The noise weight for H-infinity control	W_u_:	The control weight for Hinfinity control
W_d_:	The disturbance weight for H-infinity control	*V*(*t*) *d*(*t*)	Wind speed External disturbance of the smart system
Δ:	The uncertainty of the system	δ_M t_	The uncertainty terms for the mass matrix
δκ	The uncertainty terms for the stiffness matrix	kp, mp:	Numerical constant from zero to one
*p*	Density of the beam	*W*	Width of the smart beam
*h*	Thickness of the smart beam	*hp*, *hs*	Width of pzt patches
